# Capecitabine from X-ray powder synchrotron data

**DOI:** 10.1107/S1600536809017905

**Published:** 2009-05-20

**Authors:** Jan Rohlicek, Michal Husak, Ales Gavenda, Alexandr Jegorov, Bohumil Kratochvil, Andy Fitch

**Affiliations:** a Department of Solid State Chemistry, ICT Prague, Technicka 5, Prague, Czech Republic; b IVAX Pharmaceuticals s.r.o., R&D, Opava, Czech Republic; c Pharmaceuticals Research and Development, Branisovska 31, Ceske Budejovice, Czech Republic; d ID31 Beamline, ESRF, 6 rue Jules Horowitz, BP 220, F-38043 Grenoble Cedex, France

## Abstract

In the title compound [systematic name 5-de­oxy-5-fluoro-*N*-(pent­yloxycarbon­yl)cytidine], C_15_H_22_FN_3_O_6_, the pentyl chain is disordered over two positions with refined occupancies of 0.53 (5) and 0.47 (5). The furan ring assumes an envelope conformation. In the crystal, inter­molecular N—H⋯O hydrogen bonds link the mol­ecules into chains propagating along the *b* axis. The crystal packing exhibits electrostatic inter­actions between the 5-fluoro­pyrimidin-2(1*H*)-one fragments of neighbouring mol­ecules as indicated by short O⋯C [2.875 (3) and 2.961 (3) Å] and F⋯C [2.886 (3) Å] contacts.

## Related literature

Capecitabine is the first FDA-approved oral chemotherapy for the treatment for some types of cancer, including advanced bowel cancer or breast cancer, see: Wagstaff *et al.* (2003 ▶); Jones *et al.* (2004 ▶).
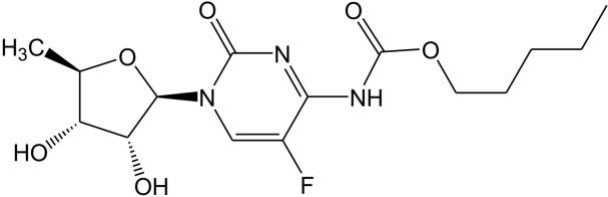


## Experimental

### 

#### Crystal data

C_15_H_22_FN_3_O_6_
*M* *_r_* = 359.35
Orthorhombic, 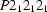

*a* = 5.20527 (2) Å
*b* = 9.52235 (4) Å
*c* = 34.77985 (13) Å
*V* = 1723.91 (1) Å^3^
*Z* = 4
Synchrotron radiation
λ = 0.79483 (4) Å
μ = 0.15 mm^−1^
*T* = 293 K
Specimen shape: cylinder
40 × 1 × 1 mm
Specimen prepared at 101 kPa
Specimen prepared at 293 K
Particle morphology: no specific habit, white

#### Data collection

ID31 ESRF Grenoble diffractometer
Specimen mounting: 1.0 mm borosilicate glass capillary
Specimen mounted in transmission mode
Scan method: step
Absorption correction: none
2θ_min_ = 1.0, 2θ_max_ = 35.0°
Increment in 2θ = 0.003°

#### Refinement

*R*_p_ = 0.055
*R*_wp_ = 0.074
*R*_exp_ = 0.036
*R*_B_ = 0.102
*S* = 2.11
Wavelength of incident radiation: 0.79483(4) Å
Excluded region(s): no
Profile function: Pseudo-Voigt profile coefficients as parameterized in Thompson *et al.* (1987 ▶), asymmetry correction according to Finger *et al.* (1994 ▶)
499 reflections
91 parameters
77 restraints
H-atom parameters not refined
Preferred orientation correction: March–Dollase (Dollase, 1986 ▶); direction of preferred orientation 001, texture parameter *r* = 1.03 (1)

### 

Data collection: *ESRF SPEC package*; cell refinement: *GSAS* (Larson & Von Dreele, 1994 ▶); data reduction: *CRYSFIRE2004* (Shirley, 2000 ▶) and *MOPAC* (Dewar *et al.*, 1985 ▶); program(s) used to solve structure: *FOX* (Favre-Nicolin & Černý, 2002 ▶); program(s) used to refine structure: *GSAS*; molecular graphics: *Mercury* (Macrae *et al.*, 2006 ▶) and *PLATON* (Spek, 2009 ▶); software used to prepare material for publication: *enCIFer* (Allen *et al.*, 2004 ▶).

## Supplementary Material

Crystal structure: contains datablocks global, I. DOI: 10.1107/S1600536809017905/cv2544sup1.cif

Rietveld powder data: contains datablocks I. DOI: 10.1107/S1600536809017905/cv2544Isup2.rtv

Additional supplementary materials:  crystallographic information; 3D view; checkCIF report

## Figures and Tables

**Table 1:** Hydrogen-bond geometry (Å, °)

*D*—H⋯*A*	*D*—H	H⋯*A*	*D*⋯*A*	*D*—H⋯*A*
N17—H171⋯O8^i^	0.860	1.956	2.797 (5)	170

Symmetry code: (i) 
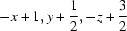
.

**Fig. 1.**
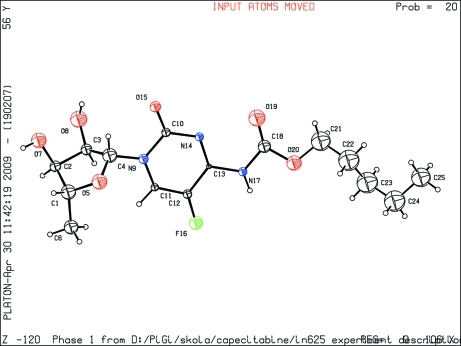
The molecular structure of capecitabine showing the atomic numbering. Displacement spheres are drawn at the 20% probability level. Only major part of the disordered pentyl chain is shown.

**Fig. 2.**
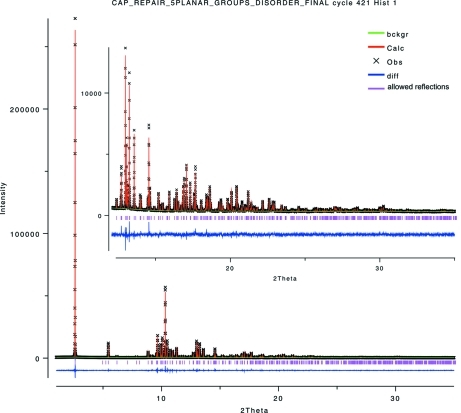
The final Rietveld plot showing the measured data (black thin-plus), calculated data (red line) and difference curve (blue line). Calculated positions of the reflections are shown by verical bars.

## supplementary crystallographic information

### Comment

Capecitabine is the first FDA-approved oral chemotherapy for the treatment for some types of cancer, including advanced bowel cancer or breast cancer (Wagstaff *et al.*, 2003; Jones *et al.*, 2004). Capecitabine is 5-deoxy-5-fluoro-*N*-[(pentyloxy)carbonyl]-cytidine and *in vivo* is enzymatically converted to the active drug 5-fluorouracil. Crystal structure determination of capecitabine was not reported yet. In this paper we report crystal structure determination of the title compound from the powder diffraction data by using synchrotron radiation.

The asymmetric unit consists of one molecule of capecitabine (Fig 1). The crystal packing is stabilized by intermolecular interactions - electrostatic interactions proved by short O···C and F···C contacts (Table 1) and N—H···O hydrogen bonds (Table 2).

### Experimental

Samples of crystalline capecitabine were prepared by two methods, *a* and *b*, respectively. Method *a*: capecitabine (10 g) was dissolved in EtOH (80 g). The solution was concentrated under reduced pressure to a residual volume of 25 ml and kept under stirring overnight. The solid was filtered off and dried at room temperature furnishing capecitabine (6 g). Method *b*: capecitabine (18 g) was dissolved in DCM (200 g) and the solution was evaporated to dryness under reduced pressure. The residue was taken up with toluene (400 g) and about 150 g of solvent were distilled off. The solution was heated up to 50°C and then allowed to 3 spontaneously cool to 25°C. After cooling to 0°C, the solid was filtered off, washed with toluene and dried at 60°C under vacuum to constant weight furnishing capecitabine (16.5 g).

### Refinement

Both crystallization procedures lead to one polycrystalline form of capecitabine. It was confirmed by measuring on X-Ray powder diffractometer PANalytical Xpert Pro, Cu *K*α radiation (λ = 1.541874 Å). Attempts to determine the structure from these data were unsuccessful probably due to flexible molecule of capecitabine and low resolution of these data. The powder obtained by the first "a" procedure was used for structure determination. X-Ray diffraction data were collected on the high resolution diffractometer ID31 of the European Synchrotron Radiation Facility. The monochromatic wavelength was fixed at 0.79483 (4) Å. Si (111) crystal multi-analyser combined with Si (111) monochromator was used (beam offset angle α = 2°). A rotating 1-mm-diameter borosilicate glass capillary with capecitabine powder was used for the experiment. Data were measured from 1.002°2θ to 34.998°2θ at the room temperature, steps scans was set to 0.003°2θ.

First 20 peaks were used by CRYSFIRE 2004 package (Shirley, 2000) to get a list of possible lattice parameters. The most probable result was selected (*a* = 5.21 Å, *b* = 9.52 Å, *c* = 34.79 Å, V = 1724 Å3, FOM (20) = 330). If 15 Å^3^ are used as an atomic volume for C, N, O and F and 5 Å3 as a volume for hydrogen atom, the approximate molecular volume is 485 Å^3^. The found volume of 1724 Å^3^ suggests that there are four molecules in the unit cell (*Z* = 4). *P2_1_2_1_2_1_* space group was selected on the basic of peaks extinction and on the basic of agreement of the Le-bail fit. The structure was solved in program FOX (Favre-Nicolin & Černý, 2002) using parallel tempering algorithm. The initial model was generated by AM1 computing method implemented in program MOPAC (Dewar *et al.*, 1985). For the solution process hydrogen atoms were removed. This model was restrained with bonds and angles restraints, automatically generated by program FOX. The refinement was carried out in *GSAS* (Larson & Von Dreele, 1994). Hydrogen atoms were added in positions based on geometry and structure was restrained by bonds and angles restraints. Five planar restraints for *sp*^2^ hybridization were used (O20/C18/O19/N17, N17/C13/N14/C12, C13/C12/F16/C11, N14/C10/O15/N9 and C4/N9/C10/C11). Due to relatively high *U*_iso_ thermal parameters of alkyl chain (C21—C25) the structure was refined with two disordered chains (C21—C25 and C21*a*—C25*a*) with occupancy factors 0.53 (5) and 0.47 (5). *U*_iso_ thermal parameters were constrained just for atoms in disordered chains by this way (C21/C21*a*, C22/C22*a*, C23/C23*a*, C24/C24*a*, C25/C25*a*). At the final stage atomic coordinates of non-hydrogen atoms were refined to the final agreement factors: *R*_p_=0.055 and *R*_wp_=0.0743. The diffraction profiles and the differences between the measured and calculated profiles are shown in Fig. 2.

### Crystal data

**Table:**

C_15_H_22_FN_3_O_6_	*D*_x_ = 1.385 Mg m^−^^3^
*M**_r_* = 359.35	Synchrotron radiation, λ = 0.79483(4) Å
Orthorhombic, *P*2_1_2_1_2_1_	µ = 0.14 mm^−^^1^
*a* = 5.20527 (2) Å	*T* = 293 K
*b* = 9.52235 (4) Å	Particle morphology: no specific habit
*c* = 34.77985 (13) Å	white
*V* = 1723.91 (1) Å^3^	cylinder, 40 × 1 mm
*Z* = 4	Specimen preparation: Prepared at 293 K and 101 kPa
*F*(000) = 760	

### Data collection

**Table:**

ID31 ESRF Grenoble diffractometer	Data collection mode: transmission
Radiation source: X-Ray	Scan method: step
Si(111)	2θ_min_ = 1.00°, 2θ_max_ = 35.00°, 2θ_step_ = 0.003°
Specimen mounting: 1.0 mm borosilicate glass capillary	

### Refinement

**Table:**

Least-squares matrix: full	91 parameters
*R*_p_ = 0.055	77 restraints
*R*_wp_ = 0.074	6 constraints
*R*_exp_ = 0.036	H-atom parameters not refined
*R*_Bragg_ = 0.102	Weighting scheme based on measured s.u.'s *w* = 1/σ(Y_obs_)^2^
χ^2^ = 4.452	(Δ/σ)_max_ = 0.05
11333 data points	Background function: Shifted Chebyschev
Excluded region(s): no	Preferred orientation correction: March–Dollase (Dollase, 1986); direction of preferred orientation 001, texture parameter r = 1.03(1)
Profile function: Pseudo-Voigt profile coefficients as parameterized in Thompson *et al.* (1987), asymmetry correction according to Finger *et al.* (1994)	

### Fractional atomic coordinates and isotropic or equivalent isotropic displacement parameters (Å^2^)

**Table:**

	*x*	*y*	*z*	*U*_iso_*/*U*_eq_	Occ. (<1)
C1	−0.0205 (8)	0.8964 (3)	0.86415 (10)	0.087 (5)*	
C2	0.0063 (7)	0.7423 (4)	0.87424 (8)	0.048 (5)*	
C3	0.0924 (6)	0.6753 (3)	0.83655 (8)	0.049 (4)*	
C4	−0.0166 (5)	0.7766 (2)	0.80775 (7)	0.081 (5)*	
O5	−0.0717 (9)	0.9090 (3)	0.82416 (10)	0.093 (3)*	
C6	0.2118 (13)	0.9888 (6)	0.87530 (18)	0.079 (4)*	
O7	−0.2355 (9)	0.6775 (5)	0.88107 (14)	0.088 (3)*	
O8	0.0594 (11)	0.5279 (3)	0.83793 (13)	0.109 (3)*	
N9	0.1175 (4)	0.79531 (18)	0.77283 (7)	0.036 (4)*	
C10	0.0276 (4)	0.73076 (17)	0.73805 (7)	0.030 (4)*	
C11	0.3307 (5)	0.87392 (18)	0.77201 (7)	0.023 (4)*	
C12	0.4772 (3)	0.90315 (14)	0.73950 (6)	0.031 (4)*	
C13	0.3691 (3)	0.83732 (13)	0.70512 (6)	0.010 (4)*	
N14	0.1675 (4)	0.75150 (16)	0.70410 (6)	0.028 (4)*	
O15	−0.1690 (5)	0.6596 (2)	0.73930 (11)	0.046 (3)*	
F16	0.6861 (5)	0.98180 (17)	0.74183 (10)	0.072 (2)*	
N17	0.4922 (3)	0.86898 (14)	0.67035 (6)	0.030 (3)*	
C18	0.4009 (4)	0.8094 (2)	0.63692 (7)	0.063 (5)*	
O19	0.2448 (4)	0.7158 (3)	0.63482 (12)	0.108 (3)*	
O20	0.5359 (5)	0.8859 (3)	0.60977 (10)	0.087 (4)*	
C21	0.491 (4)	0.8346 (15)	0.57240 (14)	0.146 (6)*	0.53 (5)
C22	0.524 (3)	0.957 (2)	0.5449 (2)	0.169 (8)*	0.53 (5)
C23	0.801 (3)	0.9940 (19)	0.5361 (5)	0.174 (9)*	0.53 (5)
C24	0.817 (4)	1.1183 (13)	0.5087 (4)	0.174 (10)*	0.53 (5)
C25	0.700 (5)	1.082 (2)	0.4695 (5)	0.143 (9)*	0.53 (5)
C21a	0.518 (5)	0.8251 (19)	0.57299 (18)	0.146 (6)*	0.47 (5)
C22a	0.680 (3)	0.9142 (19)	0.54603 (17)	0.169 (8)*	0.47 (5)
C23a	0.560 (3)	0.939 (2)	0.5068 (4)	0.174 (9)*	0.47 (5)
C24a	0.764 (5)	0.9452 (15)	0.4756 (2)	0.174 (10)*	0.47 (5)
C25a	0.925 (4)	1.079 (2)	0.4786 (7)	0.143 (9)*	0.47 (5)
H251	0.7123	1.1617	0.453	0.25*	0.53 (5)
H252	0.5245	1.0576	0.4727	0.25*	0.53 (5)
H253	0.7906	1.0057	0.4585	0.25*	0.53 (5)
H241	0.7261	1.1953	0.5195	0.25*	0.53 (5)
H242	0.9921	1.1435	0.5053	0.25*	0.53 (5)
H231	0.8866	1.0173	0.5594	0.25*	0.53 (5)
H232	0.8831	0.9152	0.5246	0.25*	0.53 (5)
H221	0.4433	1.0371	0.5559	0.25*	0.53 (5)
H222	0.4406	0.9338	0.5214	0.25*	0.53 (5)
H211	0.3216	0.7981	0.5706	0.25*	0.53 (5)
H212	0.6111	0.7627	0.5664	0.25*	0.53 (5)
H61	0.1794	1.0833	0.868	0.1*	
H62	0.2378	0.9842	0.9023	0.1*	
H63	0.361	0.9557	0.8624	0.1*	
H21	0.1249	0.7267	0.8946	0.075*	
H31	0.273	0.6894	0.8356	0.075*	
H11	−0.166	0.9315	0.8775	0.12*	
H41	−0.1786	0.7386	0.8007	0.12*	
H111	0.3869	0.9132	0.7957	0.03*	
H171	0.6224	0.9246	0.6699	0.04*	
H82	−0.0753	0.5066	0.8272	0.1*	
H72	−0.216	0.592	0.883	0.12*	
H2511	1.0505	1.0802	0.4588	0.25*	0.47 (5)
H2512	1.008	1.082	0.5029	0.25*	0.47 (5)
H2513	0.8164	1.1589	0.476	0.25*	0.47 (5)
H2411	0.874	0.8661	0.478	0.25*	0.47 (5)
H2412	0.6824	0.943	0.4511	0.25*	0.47 (5)
H2311	0.4682	1.0252	0.5072	0.25*	0.47 (5)
H2312	0.4442	0.8643	0.5013	0.25*	0.47 (5)
H2211	0.7075	1.0029	0.5578	0.25*	0.47 (5)
H2212	0.8402	0.8684	0.5424	0.25*	0.47 (5)
H2111	0.5817	0.7316	0.5736	0.25*	0.47 (5)
H2112	0.3442	0.8245	0.5647	0.25*	0.47 (5)

### Geometric parameters (Å, °)

**Table:**

C1—C2	1.515 (5)	O20—C21	1.4080 (21)
C1—O5	1.421 (5)	O20—C21a	1.4073 (21)
C1—C6	1.545 (7)	C21—C22	1.5177 (21)
C1—H11	0.950	C21—H211	0.949 (16)
C2—C3	1.525 (4)	C21—H212	0.951 (24)
C2—O7	1.422 (6)	C22—C23	1.5196 (21)
C2—H21	0.950	C22—H221	0.950 (22)
C3—C4	1.502 (4)	C22—H222	0.950 (9)
C3—O8	1.413 (4)	C23—C24	1.5219 (21)
C3—H31	0.950	C23—H231	0.950 (19)
C4—O5	1.413 (4)	C23—H232	0.951 (22)
C4—N9	1.4123 (19)	C24—H241	0.949 (19)
C4—H41	0.950	C24—H242	0.951 (20)
C6—H61	0.950	C25—C24	1.5304 (21)
C6—H62	0.950	C25—H251	0.951 (19)
C6—H63	0.950	C25—H252	0.952 (26)
O7—H72	0.820	C25—H253	0.950 (23)
O8—H82	0.820	C21a—C22a	1.5189 (21)
N9—C10	1.4352 (18)	C21a—H2111	0.950 (25)
N9—C11	1.3389 (19)	C21a—H2112	0.951 (26)
C10—N14	1.4015 (19)	C22a—C23a	1.5195 (21)
C10—O15	1.2282 (19)	C22a—H2211	0.950 (15)
C11—C12	1.3919 (19)	C22a—H2212	0.950 (21)
C11—H111	0.950	C23a—C24a	1.5233 (21)
C12—C13	1.4625 (19)	C23a—H2311	0.950 (20)
C12—F16	1.3228 (19)	C23a—H2312	0.950 (18)
C13—N14	1.3305 (18)	C24a—C25a	1.5298 (21)
C13—N17	1.4013 (19)	C24a—H2411	0.949 (21)
N17—C18	1.3783 (19)	C24a—H2412	0.952 (18)
N17—H171	0.860	C25a—H2511	0.950 (19)
C18—O19	1.2084 (20)	C25a—H2512	0.950 (27)
C18—O20	1.3839 (20)	C25a—H2513	0.950 (26)
			
O15···C12^i^	2.961 (3)	O15···C11^iii^	2.875 (3)
F16···C10^ii^	2.886 (3)		
			
C2—C1—O5	109.0 (3)	O20—C21—H212	110.1 (17)
C2—C1—C6	114.90 (20)	C22—C21—H211	110.1 (16)
C2—C1—H11	107.52	C22—C21—H212	109.9 (6)
O5—C1—C6	110.16 (20)	H211—C21—H212	109.4 (9)
O5—C1—H11	107.4	C21—C22—C23	114.26 (21)
C6—C1—H11	107.5	C21—C22—H221	108.2 (6)
C1—C2—C3	103.46 (14)	C21—C22—H222	108.2 (14)
C1—C2—O7	112.16 (19)	C23—C22—H221	108.3 (14)
C1—C2—H21	112.53	C23—C22—H222	108.3 (12)
C3—C2—O7	102.85 (18)	H221—C22—H222	109.5 (16)
C3—C2—H21	112.59	C22—C23—C24	110.85 (21)
O7—C2—H21	112.5	C22—C23—H231	109.1 (13)
C2—C3—C4	101.19 (13)	C22—C23—H232	109.1 (15)
C2—C3—O8	110.48 (18)	C24—C23—H231	109.1 (15)
C2—C3—H31	105.17	C24—C23—H232	109.1 (12)
C4—C3—O8	127.86 (19)	H231—C23—H232	109.5 (16)
C4—C3—H31	105.07	C23—C24—C25	111.26 (21)
O8—C3—H31	105.13	C23—C24—H241	109.1 (12)
C3—C4—O5	112.34 (14)	C23—C24—H242	109.0 (16)
C3—C4—N9	117.90 (12)	C25—C24—H241	109.1 (24)
C3—C4—H41	105.26	C25—C24—H242	109.0 (17)
O5—C4—N9	109.57 (17)	H241—C24—H242	109.4 (11)
O5—C4—H41	105.29	C24—C25—H251	109.6 (20)
N9—C4—H41	105.37	C24—C25—H252	109.5 (21)
C1—O5—C4	106.4 (3)	C24—C25—H253	109.6 (17)
C1—C6—H61	109.5	H251—C25—H252	109.3 (19)
C1—C6—H62	109.5	H251—C25—H253	109.5 (22)
C1—C6—H63	109.4	H252—C25—H253	109.4 (24)
H61—C6—H62	109.4	O20—C21a—C22a	107.18 (20)
H61—C6—H63	109.4	O20—C21a—H2111	110.1 (16)
H62—C6—H63	109.6	O20—C21a—H2112	109.9 (18)
C2—O7—H72	109.5	C22a—C21a—H2111	110.2 (17)
C3—O8—H82	109.47	C22a—C21a—H2112	110.1 (13)
C4—N9—C10	120.62 (14)	H2111—C21a—H2112	109.4 (6)
C4—N9—C11	119.91 (14)	C21a—C22a—C23a	114.36 (21)
C10—N9—C11	119.47 (12)	C21a—C22a—H2211	108.3 (6)
N9—C10—N14	118.71 (13)	C21a—C22a—H2212	108.2 (15)
N9—C10—O15	118.59 (15)	C23a—C22a—H2211	108.2 (19)
N14—C10—O15	122.71 (15)	C23a—C22a—H2212	108.3 (10)
N9—C11—C12	125.65 (14)	H2211—C22a—H2212	109.4 (10)
N9—C11—H111	117.16	C22a—C23a—C24a	110.99 (21)
C12—C11—H111	117.19	C22a—C23a—H2311	109.1 (20)
C11—C12—C13	111.59 (12)	C22a—C23a—H2312	109.0 (11)
C11—C12—F16	120.89 (15)	C24a—C23a—H2311	109.1 (12)
C13—C12—F16	127.52 (14)	C24a—C23a—H2312	109.2 (19)
C12—C13—N14	126.04 (12)	H2311—C23a—H2312	109.5 (16)
C12—C13—N17	115.94 (14)	C23a—C24a—C25a	111.43 (21)
N14—C13—N17	118.02 (18)	C23a—C24a—H2411	109.0 (10)
C10—N14—C13	118.29 (13)	C23a—C24a—H2412	108.9 (21)
C13—N17—C18	118.81 (13)	C25a—C24a—H2411	109.1 (26)
C13—N17—H171	120.56	C25a—C24a—H2412	109.0 (16)
C18—N17—H171	120.63	H2411—C24a—H2412	109.4 (11)
N17—C18—O19	125.88 (16)	C24a—C25a—H2511	109.5 (21)
N17—C18—O20	100.60 (15)	C24a—C25a—H2512	109.5 (21)
O19—C18—O20	133.52 (16)	C24a—C25a—H2513	109.6 (17)
C18—O20—C21	111.26 (20)	H2511—C25a—H2512	109.4 (21)
C18—O20—C21a	111.74 (20)	H2511—C25a—H2513	109.4 (23)
O20—C21—C22	107.29 (21)	H2512—C25a—H2513	109.5 (24)
O20—C21—H211	110.0 (9)		

Symmetry codes:  (i) *x*−1, *y*, *z*; (ii) −*x*+1, *y*+1/2, −*z*+3/2; (iii) −*x*, *y*−1/2, −*z*+3/2.

### Hydrogen-bond geometry (Å, °)

**Table:**

*D*—H···*A*	*D*—H	H···*A*	*D*···*A*	*D*—H···*A*
N17—H171···O8^ii^	0.860	1.956	2.797 (5)	170

Symmetry codes: (ii) −*x*+1, *y*+1/2, −*z*+3/2.
