# Capecitabine from X-ray powder synchrotron data. Corrigendum

**DOI:** 10.1107/S2056989016008252

**Published:** 2016-05-27

**Authors:** Jan Rohlicek, Michal Husak, Ales Gavenda, Alexandr Jegorov, Bohumil Kratochvil, Andy Fitch

**Affiliations:** a Department of Solid State Chemistry, ICT Prague, Technicka 5, Prague, Czech Republic; b IVAX Pharmaceuticals s.r.o., R&D, Opava, Czech Republic; c Pharmaceuticals Research and Development, Branisovska 31, Ceske Budejovice, Czech Republic; d ID31 Beamline, ESRF, 6 rue Jules Horowitz, BP 220, F-38043 Grenoble Cedex, France

## Abstract

In the paper by Rohlicek *et al.* [*Acta Cryst.* (2009), E**65**, o1325–o1326], one H atom was placed incorrectly.

## Abstract 2

Erratum to *Acta Cryst.* (2009), E**65**, o1325–o1326.

Following our powder-diffraction study of capecitabine (Rohlicek *et al.*, 2009 ▸), Malińska *et al.* (2014 ▸) published the crystal structure of the same mol­ecule based on single-crystal data. Although they modelled the wrong enanti­omer [as was pointed out by Kratochvil *et al.* (2016 ▸)], the structures are very similar after inverting the single-crystal structure, including the disordered part of the mol­ecule (Fig. 1 ▸). Since single-crystal diffraction is more sensitive to H atoms than powder diffraction, Malinska *et al.* (2014 ▸) were able to locate the H atoms directly. This indicated a different tautomeric form of capecitabine to that assumed in our study, and as they pointed out, we had therefore placed one H atom wrongly.

In our defence, in the powder study, we placed the H atoms geometrically according to a reasonable chemical structure for capecitabine, which shows the tautomeric H atom attached to the N atom of the carbamate group and the plausible formation of an inter­molecular N—H⋯O hydrogen bond. As shown by Malińska *et al.* (2014 ▸), the H atom is actually located on the N atom of the pyrimidine ring (Fig. 2 ▸), thereby forming an intra­molecular N—H⋯O link.

With respect to the fact that structure solution from powder diffraction data is based on the proposed molecular structure, readers should beware of the incorrectly placed H atom in Rohlicek *et al.* (2009 ▸) and they should be also beware of the wrong enantiomer in a single-crystal study of Malińska *et al.* (2014 ▸).

## Figures and Tables

**Figure 1:**
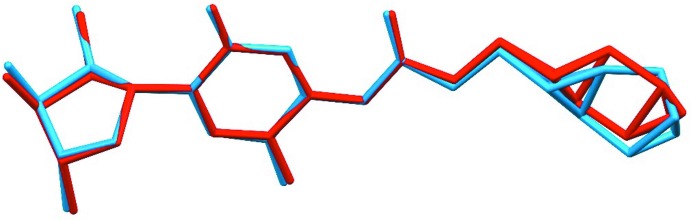
Overlay of the capecitabine mol­ecular structures arising from powder diffraction (blue) and from single-crystal diffraction data (red). Only non-H atoms are shown for clarity.

**Figure 2:**
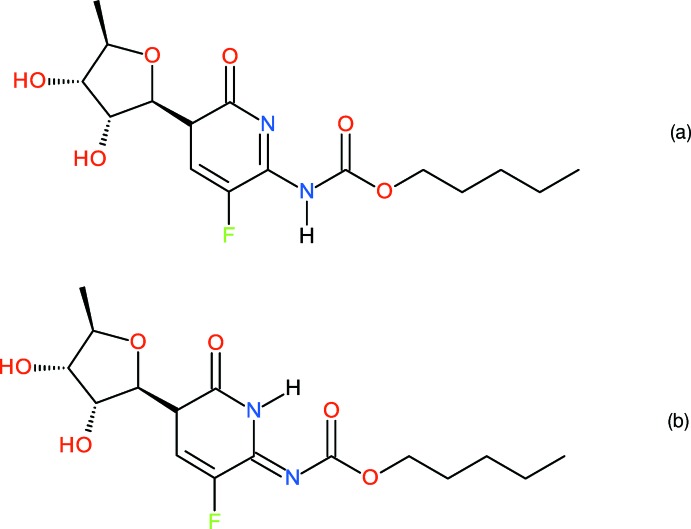
Schemes for the tautomeric forms of capecitabine (*a*) assumed in the powder-diffraction study and (*b*) established in the single-crystal study of Malinska *et al.* (2014 ▸).

## References

[bb1] Kratochvil, B., Husak, M., Korotkova, E. I. & Jegorov, A. (2016). *Chem. Listy*, **110**, 40–47.

[bb2] Malińska, M., Krzecyński, P., Czerniec-Michalik, E., Trzcińska, K., Cmoch, P., Kutner, A. & Woźniak, K. (2014). *J. Pharm. Sci.* **103**, 587–593. 10.1002/jps.23831 24382662

[bb3] Rohlicek, J., Husak, M., Gavenda, A., Jegorov, A., Kratochvil, B. & Fitch, A. (2009). *Acta Cryst.* E**65**, o1325–o1326. 10.1107/S1600536809017905 PMC2969674 21583180

